# A cross-sectional study on serum high-sensitivity C-reactive protein level and shift work among reproductive age women

**DOI:** 10.22088/cjim.10.4.402

**Published:** 2019

**Authors:** Maryam Nikpour, Aram Tirgar, Mahmod Hajiahmadi, Abbas Ebadi, Fatemeh Ghaffari, Akram Hosseini, Behzad Heidari

**Affiliations:** 1 Student Research Committee, Health Research Institute, Babol University of Medical Sciences, Babol, Iran; 2 Social Determinants of Health Research Center, Health Research Institute, Babol University of Medical Sciences, Babol, Iran; 3 Non Communicable Pediatric Diseases Research Center, Health Research Institute, Babol University of Medical Sciences, Babol, Iran; 4 Behavioral Sciences Research Center, Life style institute, Nursing Faculty, Baqiyatallah University of Medical Sciences, Tehran, Iran; 5 Nursing Faculty, Baqiyatallah University of Medical Sciences, Tehran, Iran; 6 Nursing Care Research Center, Health Research Institute, Babol University of Medical Sciences, Babol, Iran; 7 Clinical Research Development Center, Shahid Beheshti Hospital, Health Research Institute, Babol University of Medical Sciences, Babol, Iran; 8 Mobility Impairment Research Center, Health Research Institute, Babol University of Medical Sciences, Babol, Iran

**Keywords:** Shift work, Reproductive age, High-sensitivity C-Reactive Protein, Cardiovascular disease, Women

## Abstract

**Background:**

Among the inflammatory factors, high-sensitivity C-reactive protein (hs-CRP) is one of the strongest predictors of cardiovascular disease. This study aimed to evaluate the relationship of serum hs-CRP level with shift work among reproductive age women.

**Methods:**

This cross-sectional study was conducted from September 2017 to May 2018 in three cities in Mazandaran Province, North of Iran. Through purposive sampling, 350 women (172 shift workers and 178 day workers) were recruited. The guideline recommended by the American Heart Association and the Centers for Disease Control and Prevention was used to interpret the result of hs-CRP measurement as the following: less than 1 µg/ml: low CVD risk; 1–3 µg/ml: moderate CVD risk; and more than 3 µg/ml: high CVD risk. The data were analyzed using the independent-sample t and the chi-square tests as well as the logistic regression analysis.

**Results:**

11.1% of participants had a serum hs-CRP level of more than 3 µg/ml. This rate among shift workers was significantly greater than day workers (15.6% vs. 7.0%; p=0.012). After adjusting the effects of potential confounders shift work significantly increased the odds of serum hs-CRP level of more than 3 µg/ml by 2.45 times (OR=2.45, 95% CI: 1.01–5.93, p=0.047).

**Conclusion:**

Shift work is a significant predictor of high serum hs-CRP level probably due to its association with sleep problems and occupational stress. Improving the knowledge of female shift workers about the importance of quality sleep can reduce their CVD risk and improve their health.

C-reactive protein (CRP) is a typical acute phase protein, which has increased in response to injury as well as systemic inflammation (1). CRP is a marker for infection and synthesized mainly by liver in response to proinfammatory cytokines (i.e. interleukin (IL)-1, IL-6, IL-17 and tumor necrosis factor-α) (2). High-sensitivity assay technique can detect CRP with a range of 0.01 to 10 mg/ l (3). hs-CRP is the most sensitive inflammatory marker and the strongest predictor of cardiovascular disease (CVD) (4, 5). Increases in serum hs-CRP level increase the risk of CVD by 2–5 times (4), diabetes mellitus (3), hypertension (6), depression (7) and cancer (8). Some studies have shown serum hs-CRP increases in shift workers (9, 10). Shift work is defined as the work in unusual work hours, i.e. at the time interval between 18:00 and 07:00.

The exact mechanism of greater risk of hs-CRP among shift workers is still unknown. Moreover, staying awake overnight causes disturbances in circadian rhythm and sleep-wake cycle (11). Circadian rhythm disturbances are associated with increase in the levels of proinflammatory cytokines (11) such as interleukin-1, interleukin-6 and tumor necrosis factor alpha (12). Interleukin-1 and interleukin-6 are the most significant factors behind the production of hs-CRP in the liver (13). Increases in the level of tumor necrosis factor alpha are also associated with increases in the level of serum hs-CRP (14).

Previous studies into the relationship of shift work and serum hs-CRP level reported contradictory results. For instance, a study among the workers of a coca processing company in Ghana found that serum hs-CRP level among shift workers was significantly higher than day workers (9). Two other studies among 1877 Finish airline-company employees and 96 Iraqi nurses also reported the same finding (10, 15). However, studies on Japanese workers and Iranian female shift work nurses found that shift work was not an independent significant predictor of serum hs-CRP level (13, 16). Their contradictory results may be related to work schedule, monthly night work (17), occupational stress (18) and gender (19). 

 Besides their contradictory results, most previous studies in this area were conducted on men, some certain occupational groups, and workers with different age, and no study had yet been conducted on reproductive women. Increase hs-CRP and related consequences among reproductive women is very important, because it has negative effects on reproduction, pregnancy, delivery, and fetal health and development (20). Thus, the present study was undertaken to fill this gap. This study will identify health problems of women in reproductive age; and it could be useful for planning prevention strategies in these employee women. Thus, the present study was undertaken to fill this gap. The aim of the study was to evaluate the relationship of serum hs-CRP level with shift work among reproductive age women.

## Methods

**Design:** This two-group cross-sectional correlational study was conducted from September 2017 to May 2018 in the three cities of Amol, Babol, and Qhaemshahr, in Mazandaran Province, North of Iran. Study population: Participants were female shift workers and day workers who were purposively selected from hospitals affiliated to Babol University of Medical Sciences, Babol, Iran, nursing homes, rehabilitation centers, and textile and sewing mills. Selection criteria were an age of 20–45, to be married, work experience of two years or more, no history of using contraceptive or estrogenic agents, no history of rheumatic disorders or infectious diseases in the past one week, no history of CVD, and a self-reported blood pressure of less than 140/90 at the beginning of employment. Participants were excluded if they voluntarily withdrew from the study. The sample size was calculated based on the results of an earlier study (15) the association between shift work and hs-CRP level which reported the difference between means score of hs-CRP in shift work and day work 0.3, and variance (1.8) ^2^ (hs-CRP in the direction for shift work). Accordingly, with a confidence interval level of 95%, a power test of 80%, and a standardized effect size of 15% sample size was estimated to be 326, and considering probable withdrawal from the study, 350 female workers were recruited. 

**Data collection:** Besides serum hs-CRP level, we assessed participants’ anthropometric indices as well as their conventional cardiovascular risk factors such as hypertension, hyperlipidemia, and diabetes mellitus in order to provide more reliable data about the effects of shift work on serum hs-CRP level. 

***Biomarkers assessment:*** In order to measure the serum levels of hs-CRP, triglyceride, high-density lipoprotein (HDL) and fasting blood sugar (FBS), a five-milliliter blood sample was obtained from each participant at 07:00–09:00 after a fasting period of 12–14 hours. Blood sampling was performed by the first author. Serum level of hs-CRP was measured using an enzyme-linked immunosorbent assay kit (Monobind Inc., USA) and a Hyperion ELISA reader device (USA). Moreover, HDL, triglyceride, and FBS levels were measured using Pars Azmoon kits (Pars Azmoon Inc., Tehran, Iran) and a biochemistry analyzer (Microsoft S800, Ireland). All laboratory tests were performed by one laboratory technician and in a hospital laboratory affiliated to Babol University of Medical Sciences, Babol, Iran. According to the guideline recommended by the American Heart Association and the Center for Disease Control and Prevention, the results of hs-CRP measurement were categorized as follows: less than 1 µg/ml: low CVD risk; 1–3 µg/ml: moderate CVD risk; and more than 3 µg/ml: high CVD risk (21). Moreover, triglyceride levels of more than 150 mg/dL, FBS levels of more than 110 mg/dL, and HDL levels of less than 40 mg/dL were considered abnormal (22). 

**Anthropometric assessment:** Participants’ weight and height were measured at the upright position with no shoes and with minimum possible clothing covering the whole body. Weight and height measurements were done using an analogue weight scale (MW84, EmsiG Co., Gm-bH) and a wall-mounted plastic tape, respectively. Then, body mass index (BMI) was calculated and interpreted as the following: 18.5–25: normal; 25–30: overweight; and more than 30: obese. Waist circumference was also measured at the midway between the lowest rib edge and the iliac crest using a plastic tape. Anthropometric assessment was done by the first author. 

**Blood pressure measurement:** An analogue sphygmomanometer (EmsiG Co., Gm-bH) was used to measure blood pressure from the right arm and after a fifteen-minute rest in the sitting position. A systolic blood pressure of more than 140 mm Hg or a diastolic blood pressure of more than 90 mm Hg was considered as hypertension. Participants who were taking antihypertensive medications were also classified as hypertensive (23). Blood pressure was measured by the first author.

***Shift work assessment:*** Participants’ work schedule was classified as either shift work or day work. Those participants who did morning or evening work shifts or both and did less than five night shifts per month were classified as day workers, while those who did at least five night shifts per month were classified as shift workers. 

***Assessment of demographic, occupational, and medical characteristics: ***A questionnaire was used to assess participants’ demographic, occupational, and medical characteristics. The items of this questionnaire were on age, educational level, place of residence, family income level, number of children, work experience, monthly work hours, organizational position, workload, occupational stress, and CVD affliction. Workload and occupational stress were separately assessed using a question with a five-point Likert scale, the possible points of which ranged from “Light” to “Heavy” and from “Very low” to “Very high”, respectively. The history of CVD was also assessed through a self-report yes/no question. 

***Assessment of health-related behaviors:*** On cigarette smoking and alcohol consumption, each was assessed via a yes/no question. Sleep quality was also assessed via a question about the average sleep hours per day (responded as “Less than six hours” or “More than six hours”), a yes/no question about hypnotic use, a yes/no question about sleep adequacy, and a question about insomnia (responded on a four-point Likert scale as either “Never”, “Rarely”, “Often”, or “Always”). For physical activity assessment, participants who did thirty-minute physical activity at least thrice weekly were classified as physically active and those with lower levels of physical activity were classified as physically inactive. 

**Statistical data analysis:** Data were analyzed via the SPSS for Windows program (v. 16.0. Chicago, IL, USA). The independent-sample *t* and the chi-square tests were performed to compare shift workers and day workers respecting their demographic, occupational, and medical characteristics and health-related behaviors. Moreover, the logistic regression analysis was employed to assess the effects of shift work on the odds of high serum hs-CRP level adjusted for potential confounders. The level of significance was set at less than 0.05. 

**Ethical considerations:** The Ethics Committee of Babol University of Medical Sciences, Babol, Iran, approved this study (code: MUBABOL.HRI.REC.1395.58). Participants were granted the right to voluntarily withdraw from the study and then, their personal written informed consents were gotten. 

## Results

Participants’ demographic, occupational, and medical characteristics and health-related behaviors

Among the 350 participating female workers, twelve shift workers and six day workers were excluded due to voluntary withdrawal, reluctance to give a blood sample, incomplete answering to the study questionnaire, or inaccessibility during the study. Consequently, final data analysis was conducted on the data collected from 332 participants, i.e. 160 shift workers and 172 day workers. There were no statistically significant differences between participants and those who were excluded with respect to their demographic, occupational, and medical characteristics and health-related behaviors (p>0.05). 

The means of participants’ age and work experience were 36.19±5.19 and 12.27±5.77 years, respectively. More than two thirds of them had university degrees (71%) and lived in urban areas (83%) and around two thirds of them were healthcare providers (64%). There were no significant differences between shift workers and day workers with respect to their age, work experience, educational level, and employment status (p>0.05; table 1). More than half of the participants had heavy or very heavy workload, (61.9%) experienced high occupational stress (52.6%), and 11.8% of them suffered from CVD. Occupational stress and CVD rate among shift workers were significantly higher than day workers (p<0.01; table 1). Most participants were physically inactive (91.7%) and were either overweight or obese (65.7%). Only 1.2% of them reported cigarette smoking and none of them reported alcohol consumption. There were no significant between-group differences respecting participants’ physical activity status, BMI, cigarette smoking, and alcohol consumption (p>0.05; tables 1 and 2). Almost half of the participants reported sleep inadequacy (47.6%) and most of them reported sleeping more than six hours a day (84.8%). Shift workers reported significantly greater sleep inadequacy and lower sleeping hours a day than day workers (p<0.05; table 2). 

**Table1:** Participants’ demographic, occupational, and medical characteristics

**Characteristics**		**Total**		**Group**		**P value**
				**Shift workers**	**Day workers**	
Age (Year) (Mean±SD)		36.19±5.29		36.37±5.47	36.37±5.47	0.52 a
Work experience (Year) (Mean±SD)		12.27±5.77		11.74±5.27	12.77±5.77	0.22 a
Monthly work hours (Mean±SD)		185.90±22.21		192.61±23.33	179.12±18.76	<0.001 a
		**N**	**%**	**N (%)**	**N (%)**	
Age (Year)	20–29	35	10.6	19 (11.9)	16 (9.4)	0.19 b
	30–39	196	59.4	87 (54.4)	109 (64.1)	
	40–45	99	30	54 (33.8)	45 (26.5)	
Educational level	Bachelor’s	234	70.9	112 (70.9)	122 (70.9)	0.97 b
	Diploma	51	15.5	25 (15.8)	26 (15.1)	
	Below diploma	45	13.6	21 (13.3)	24 (14)	
Place of residence	Urban areas	55	16.7	30 (19.0)	25 (14.5)	0.27 b
	Rural areas	275	83.3	128 (81.0)	147 (85.0)	
Family income	Sufficient	123	37.20	48 (30.4)	71 (43.9)	0.032 b
	Moderately sufficient	152	46.2	79 (50.0)	73 (42.7)	
	Insufficient	54	16.41	31 (19.6)	23 (13.5)	
Physical activity	Active	143	38.3	61 (33.3)	82 (43.2)	0.51 b
	Inactive	230	61.7	122 (66.7)	108 (56.8)	
Cigarette smoking	Yes	4	1.2	158	170	0.92 c
	No	328	98.81	2 (1.2)	2 (1.2)	
Employment status	Healthcare provider	273	70.7	136 (69.4)	137 (72.1)	0.17 b
	Mother aid or nurse aid	61	15.7	38 (19.4)	23 (12.1)	
	Laborer	34	8.8	15 (7.7)	19 (10.0)	
	Service staff	18	4.7	7 (3.6)	11 (5.8)	
Workload	Light to moderate	123	38.1	51 (33.9)	72 (42.9)	0.14 b
	Heavy	135	41.8	68 (43.9)	67 (39.9)	
	Very heavy	65	20.1	36 (23.2)	29 (17.3)	
Occupational stress	Low	38	11.7	18 (11.5)	20 (11.8)	0.004 b
	Moderate	116	35.7	74 (43.8)	74 (43.8)	
	High	171	52.6	96 (61.5)	75 (44.4)	
Cardiovascular problem	Yes	38	11.8	24 (15.6)	14 (8.3)	0.042 b
	No	285	88.2	130 (88.2)	155 (91.7)	

a: The results of the independent-sample* t *test; b: The results of the Chi-square test; c: The results of the Fisher’s exact test

**Table2:** Participants’ anthropometric indices, biomarkers, and sleep-related parameters

**Variables**		**Total**		**Group**		**P value**
				**Shift workers**	**Day workers**	
		**N**	**%**	**N (%)**	**N (%)**	
Insomnia	Rarely	152	47.5	57 (37.3)	95 (56.9)	0.002^a^
	Mostly	118	36.9	66 (43.1)	52 (31.1)	
	Always	50	15.6	30 (19.6)	20 (12)	
Hypnotic use	Yes	46	14.3	21 (13.7)	25 (14.9)	0.76 ^a^
	No	275	85.7	132 (86.3)	143 (85.7)	
Aadequacy of sleep	Yes	167	52.4	71 (46.4)	96 (57.8)	0.041^ a^
	No	152	47.6	82 (53.6)	70 (42.2)	
Average daily sleep hours	≤ 6	41	15.2	32 (23.7)	9 (6.7)	0.<001 ^a^
	> 6	228	84.8	103 (76.3)	125 (93.3)	
Body Mass Index (kg/m^2^)	18.5–25	114	34.3	49 (30.6)	65 (37.8)	0.081 ^a^
	25–30	145	43.7	80 (50.0)	65 (37.8)	
	< 30	73	22.0	31 (19.4)	24 (24.4)	
Waist circumference (cm)	< 88	102	30.7	46 (28.8)	56 (32.6)	0.45 ^a^
	≥ 88	230	69.3	114 (71.3)	116 (67.4)	
Blood pressure (mm Hg)	< 140/90	324	97.6	155 (96.9)	169 (98.3)	0.48 ^b^
	≥ 140/90	8	2.4	5 (3.1)	3 (1.7)	
Fasting blood sugar (mg/dL)	< 110	328	98.8	157 (98.1)	171 (99.4)	0.35 ^a^
	≥ 110	4	1.2	3 (1.9)	1 (0.6)	
High-density lipoprotein (mg/dL)	> 50	266	19.9	137(85.6)	129(75.0)	0.015a
	≥ 50	66	80.1	23(14.4)	43(25.0)	
Triglyceride (mg/dL)	< 150	274	82.5	131 (81.9)	143 (83. 1)	0.76 a
	≥ 150	58	17.5	29 (18.1)	29 (16.9)	
hs-CRP μg/ml	≤ 3 (Low and Moderate risk)	295	88.9	135 (84.4)	160 (93.0)	0.012 a
	> 3 (High risk)	37	11.1	25 (15.6)	12 (7.0)	

a: The results of the Chi-square test; b: The results of the Fisher’s exact test

**Participants’ hs-CRP, biomarkers, and blood pressure: **The total mean of serum hs-CRP level was 1.27±1.57 µg/ml. This value among shift workers and day workers was respectively 1.45±1.80 and 1.11±1.32, with a statistically significant between-group difference (p=0.05). Moreover, 11.1% of participants had a serum hs-CRP level of more than 3 µg/ml. This rate was 15.6% among shift workers and 7.0% among day workers, with a significant between-group difference (p=0.012; table 2). Only 2.4% of participants suffered from hypertension and respectively 1.2%, 17.5%, and 63.6% of them had abnormal serum levels of FBS, triglyceride, and HDL. The between-group differences respecting hypertension rate and serum levels of FBS, triglyceride, and HDL were not statistically significant (p>0.05; table 2). The relationship of serum hs-CRP level with shift work The results of the logistic regression analysis illustrated that after adjusting the effects of the potential confounders (namely age, educational level, BMI, physical activity, occupational stress, work experience, work hours, and employment status), shift work significantly increased the risk of high hs-CRP by 2.45 times (OR=2.45, 95% CI: 1.01–5.93, p=0.047); table 3).

**Table 3:** The results of the logistic regression analysis to predict high serum hs-CRP level based on work schedule adjusted for potential confounders

**Model 5**	**Model 4**	**Model 3**	**Model 2**	**Unadjusted**	**Model** **Schedule**
1	1	1	1	1	Day work
2.45 (1.01–5.93)	2.28 (1.00–5.23)	2.07 (0.98–4.38)	2.48 (0.1.20–5.12)	2.46 (1.19–5.10)	Shift work

Model 1: Single factor logistic regression

Model 2: Adjusted for age

Model 3: Adjusted for Model 2 plus educational level and family income

Model 4: Adjusted for Model 3 plus BMI and physical activity

Model 5: Adjusted for Model 4 plus work experience, work hours, employment status, and occupational stress Reference group was day work

## Discussion

This study aimed to evaluate the relationship of serum hs-CRP level with shift work among reproductive age women. Findings showed that 11.1% of participants had a serum hs-CRP level of more than 3 µg/ml. Similarly, a study on Australian women who aged 25–39 reported that 15.7% of them had a serum hs-CRP level of more than 3 µg/ml (24). However, several other studies reported much higher rates of high serum hs-CRP level. For instance, this rate was 20.9% and 33.3% among Iranian women (16, 25) and 48% among American women who aged more than 45 (26). The lower rate of high serum hs-CRP level in the present study can be attributed to our participants’ age and educational level so that while an age of 20–45 was one of the selection criteria of the present study, those studies were conducted on women with different ages including women over 45. Studies showed that serum hs-CRP level increases with age (27, 28). Moreover, 70% of our participants had university degrees. Higher educational level is associated with greater awareness of CVD risk factors and hence, lower risk of CVD development (29). Another justification behind the lower rate of high serum hs-CRP level in the present study may be the lower rates of hypertension, cigarette smoking, and hyperglycemia among study participants. Serum hs-CRP level positively correlates with cigarette smoking rate (30, 31), blood pressure (23), and FBS level (32). 

Study findings also showed that the rate of high serum hs-CRP level among shift workers was significantly greater than day workers. Several studies in Finland (11), Ghana (9), Korea (33), and Iraq (15) reported the same. Pavanello et al. and Heydarikhayat et al. confirmed this result (10, 34). However, two studies on Japanese workers (13) Omani nurses (35) and Netherlands health care workers (36) found that shift work was not a significant factor behind hs-CRP variations. One justification for the significant relationship of shift work with high serum hs-CRP level may be circadian misalignment due to shift work (37). A study which simulated the workplace of shift workers and day workers in a laboratory setting reported that circadian misalignment among shift workers significantly increased the rate of high serum hs-CRP level by 11% (37). Circadian misalignment increases the production of proinflammatory cytokines such as interleukin-1, interleukin-6 (12), and tumor necrosis factor alpha (12). Interleukin-1 and interleukin-6 are among the most significant factors behind the production of hs-CRP in the liver (13). Increases in the level of tumor necrosis factor alpha are also associated with increases in the level of serum hs-CRP (14).

Another justification for the significant relationship of shift work with high serum hs-CRP level may be the higher rate of sleep problems (such as insomnia and sleep inadequacy) among our shift worker participants. Some studies reported that sleep disturbances in shift workers is a factor risk to increase hs-CRP (17, 33), while, Puttonen et al. did not confirm these results (11). Sleep disturbances alter the expression of clock genes in the central and peripheral parts of the body clock center and thereby, increase inflammatory responses and serum levels of inflammatory factors such as hs-CRP(26). A study reported that the risk of high serum hs-CRP level among people with sleep duration of less than 5.5 hours a day was 2.2 times more than those with longer sleep duration (38). 

The other justification for the higher serum level of hs-CRP among shift workers in our study may be the fact that their occupational stress was significantly greater than their day worker counterparts. Xu W et al. in their study among Chinese workers also showed that occupational stress significantly increases risk of high hs-CRP 18). Previous studies showed that occupational stress among shift workers significantly increased serum cortisol level (39, 40). Through a complex process, cortisol stimulates the production of those cellular and molecular proteins which contribute to inflammation and thereby, increases the levels of inflammatory factors (41) such as hs-CRP. A study reported that through increasing inflammatory factors in vascular endothelium, both acute and chronic stress may induce atherosclerosis and result in CVD (42). 

**Limitations:** This study was conducted using a cross-sectional design. Cohort studies are recommended to more carefully assessing the relationship of shift work with serum hs-CRP level. Moreover, rheumatic disorders among participants were assessed through the self-report method. Some participants might have been afflicted by these disorders but were unaware of such affliction. Therefore, future studies are recommended to screen participants respecting rheumatic disorders through clinical and laboratory methods. 

In addition, study participants were reproductive women who worked as either shift workers or day workers. Assessment of unemployed and menopausal women can provide more reliable information about the effects of age and employment on serum hs-CRP level. The other study limitation was the small number of women who worked as laborer, mother aid, and nurse aid due to the small number of rehabilitation centers, nursing homes, and industrial mills with female workers in the study setting. 

In conclusion, this study concludes that shift work is a significant predictor of high serum hs-CRP level among shift workers probably due to the higher prevalence of sleep problems and occupational stress among them. Strategies such as providing female shift workers with counseling services in order to improve their knowledge about the importance of quality sleep and healthy eating can reduce their serum hs-CRP level, improve their health status, and thereby, reduce CVD risk among them. 
